# Pharmacogenetics of SSRIs and Sexual Dysfunction

**DOI:** 10.3390/ph3123614

**Published:** 2010-12-15

**Authors:** Liana Osis, Jeffrey R. Bishop

**Affiliations:** Department of Pharmacy Practice, College of Pharmacy, University of Illinois at Chicago, 833 S. Wood St. Rm. 164 (M/C886), Chicago, IL 60612, USA

**Keywords:** SSRI, sexual dysfunction, polymorphism, serotonin, glutamate, drug metabolism, depression

## Abstract

Sexual dysfunction (SD) is a common and disconcerting side effect of selective serotonin reuptake inhibitors (SSRIs) that often influences a patient’s desire to continue long-term antidepressant treatment. Studies specifically assessing changes in sexual well-being over time illustrate that the incidence of sexual side effects from SSRIs ranges from 20% to 70%, depending on the characteristics of the study sample assessed. Developing strategies to predict who may be at the highest risk for adverse changes in their sexual well-being is an important step in improving the quality of life and treatment of patients who require antidepressant therapy. Pharmacogenetic studies of SSRI-associated SD have identified associations between serotonin and glutamate system genes with aspects of SD. The results of studies investigating genetic variations in drug metabolism enzymes and their relationships to antidepressant-associated adverse effects have been mixed. Continued efforts to characterize the relationships between genetic markers and antidepressant outcomes, and to translate this knowledge to patient care, have the potential to significantly improve the empiric selection of antidepressant agents and to minimize the risk for intolerable side effects.

## 1. Introduction

Approximately 19 million Americans suffer from depression every year, with estimates that 16.2% of the United States population will experience a major depressive episode in their lifetime [1]. Selective serotonin reuptake inhibitors (SSRIs) such as citalopram, fluoxetine, fluvoxamine, escitalopram, paroxetine, and sertraline are common choices as first-line antidepressants. The efficacy of these drugs is superior to placebo and comparable to other classes of antidepressants in treating patients with major depression [2,3]. While the response rates of these drugs in randomized controlled efficacy trials is ~50-70% [3], effectiveness studies highlight that use in “real world” subjects yields lower response (47%) and remission (28-33%) rates [4]. 

Side effects and medication non-adherence to therapies are two reasons for inadequate responses to antidepressant agents. Approximately 20% of patients will discontinue their antidepressant medications [5]. The total number of adverse effects is a significant predictor of medication non-adherence; 36% of patients who stop their medication do so because of adverse effects [5,6]. Sexual dysfunction (SD) is commonly observed during SSRI therapy, occurring in approximately20-70% of patients taking an SSRI [7,8,9]. These sexual side effects are particularly disconcerting to patients because they are persistent and generally do not abate like headache, nausea, insomnia, diarrhea, and other early onset side effects, which generally dissipate after the first few weeks of therapy. Only 10-15% of patients experiencing SSRI-associated SD report significant improvements after 6-12 months [9,10,11]. However, the occurrence of sexual side effects and the specific domain(s) of the sexual experience affected differ greatly from patient to patient. This indicates that there are likely underlying genetic factors that may influence an individual’s response to a specific antidepressant therapy.

Pharmacogenetic studies of antidepressant-associated SD are relatively new, with the oldest published results dating back to 2002. The high prevalence of SSRI-associated sexual side effects and the negative impact they may have on drug therapy underscores the importance of conducting investigations to identify and characterize variables associated with these undesirable outcomes. In the context of a growing cadre of antidepressants with differing mechanisms of action and metabolic pathways, the therapeutic potential of a pharmacogenetic test to identify risk for SSRI-associated SD, if developed, is extremely high. To this end, this review will summarize the results of pharmacogenetic studies of SD in patients taking SSRIs. The results to date implicate gene variants relating to the pharmacokinetics as well as the pharmacodynamics of these medications and include variants in genes coding for drug metabolizing enzymes, as well as serotonin signaling, glutamate activity, and neurotrophic factors.

## 2. Experimental Section

### 2.1. Methods

A literature review was completed to identify pharmacogenetic studies of SSRI-associated SD. Medline via PubMed, Excerpta Medica Database (EMBASE), the American Psychological Association’s PsycINFO database, and the Cochrane Library were searched using the following terms: “antidepressant AND pharmacogenetics,” “antidepressant AND polymorphism”, “citalopram AND pharmacogenetics,” “fluoxetine AND pharmacogenetics,” “fluvoxamine AND pharmacogenetics,” “escitalopram AND pharmacogenetics,” “sertraline AND pharmacogenetics,” and “paroxetine AND pharmacogenetics,” citalopram AND polymorphism,” “fluoxetine AND polymorphism,” “fluvoxamine AND polymorphism,” “escitalopram AND polymorphism,” “sertraline AND polymorphism,” and “paroxetine AND polymorphism”. The results of these queries were searched for research studies investigating genetic predictors of outcomes from SSRI treatment. These studies were further narrowed to those specifically assessing measures of SD and relationships with genetic markers which are summarized in this review.

## 3. Results

### 3.1. Hypothesized Mechanisms of SSRI-Associated Sexual Dysfunction

The sexual experience can broadly be divided into three phases. Stage 1: interest and desire (libido); Stage 2: physiologic arousal; and Stage 3: orgasm. Neurotransmitters and hormones are believed to influence SSRI-associated SD [12,13]. Dopamine, serotonin (5HT), testosterone, and estrogen influence sexual interest and desire (libido). Nitric oxide, acetylcholine, and 5HT are important modulators of physiological sexual arousal. Finally, norepinephrine and 5HT play important roles in orgasm. Recent evidence suggests that additional neurotransmitters such as glutamate may also be involved with sexual physiology [14]. While the importance of other neurobiological contributors to the sexual experience should not be underestimated, the current review will focus on the mechanisms hypothesized to be associated with SSRI-associated sexual side effects and the pharmacogenetic studies of these factors. 

SSRIs inhibit 5HT reuptake via antagonism of the serotonin transporter which increases synaptic 5HT [15]. Although the underlying mechanisms are not completely elucidated, it is generally accepted that 5HT serves to diminish sexual function [13,16] and dopamine enhances sexual function [17]. The specific effects of serotonin on the sexual experience may result from signaling through specific subtypes of 5HT receptors. Relevant to the pharmacology of antidepressant agents, it is thought that stimulation of 5HT2A receptors has negative effects on sexual function [12,13,17,18]. Stimulating 5HT pathways in the raphe nuclei, which project down the spine as well as into the brain, are hypothesized to negatively influence the three stages of the sexual experience [12,13,17,18]. Consistent with this hypothesis, pharmacological stimulation of 5HT2 and 5HT3 receptors impairs sexual function in rodents [16]. Furthermore, in humans, antidepressant drugs which increase 5HT signaling (e.g. SSRIs) in the absence of 5HT2A antagonism are commonly associated with sexual side effects while agents which possess 5HT2A antagonist activity (e.g. mirtazapine and nefazodone) have a relatively low likelihood of inducing sexual side effects [8]. Until recently, the influence of genetic variation in the serotonin system and other pathways on changes in sexual function during antidepressant treatment has remained relatively unstudied. However, initial pharmacogenetic studies provide further insight into these relationships and are informative for the development of future research strategies to investigate these mechanisms and genetic risk factors. 

### 3.2. Pharmacogenetic Studies of SSRI-Associated Sexual Dysfunction: Genetic Variation in Drug Metabolism Genes

Studies indicate that SSRI-associated SD is dose-related, and that SD is common among allSSRIs [8,19]. Thus it is reasonable to hypothesize that genetic variation in enzymes essential for SSRI metabolism which result in reduced enzymatic activity may increase systemic exposure to an antidepressant and increase the likelihood of experiencing sexual side effects. 

The CYP450 isoenzyme family is essential for the metabolism of many SSRIs. Specifically, CYP2D6 and CYP2C19 are two subfamilies in which inherited genetic variability exists which confer a “poor metabolizer” state and therefore may influence the likelihood of experiencing sexual side effects. Variation in the metabolic capacity of one of these enzymes, CYP2D6, has been studied for associations with sexual side effect outcomes, and may be stratified to four phenotypic classes:1) ultrarapid metabolizers (UM) where there is an increase in enzymatic activity due to gene duplication; 2) extensive (normal) metabolizers (EM); 3) intermediate metabolizers (IM) who possess genetic variation which diminishes but does not eliminate enzymatic function; and 4) poor metabolizers (PM), who possess genetic variants which essentially eliminate CYP2D6 function. Approximately 5-10% of Caucasian individuals are PMs while the prevalence of PM status in other populations is generally lower [20].

Two studies have attempted to investigate the relationship between genetic variants in the *CYP2D6* gene and SSRI-associated SD. The overall goal of the first investigation was to characterize predictors of SD in subjects treated with the SSRI paroxetine [21], which is predominantly metabolized by CYP2D6 [19]. Of the 52 participants in the parent investigation, 30 gave their consent to participate in an exploratory study of CYP2D6 metabolizer status and paroxetine-associated sexual dysfunction. Participants enrolled in this cross-sectional study had already been treated with paroxetine (meandose = 20.8 ± 5.6 mg/d) for a duration ranging from 42-300 days. A total of 21 women and nine men (ages 23-56 years) were phenotyped for CYP2D6 metabolic status using standard dextromethorphan measures and categorized to either EM or PM metabolizer status. Genotyping for *3, *4, *5, and *6 variants of the *CYP2D6* gene was also completed. The Arizona Sexual Experience (ASEX) scale was used to assess sexual well-being. The ASEX scale measures overall sexual functioning and includes specific items to assess libido, arousal, and penile erection/vaginal lubrication. In the 30 participants participating in the pharmacogenetics portion of the study, 63% (19 out of 30) reported SD. In this study, none of the 30 participants investigated possessed a PM *genotype*. The genotype distribution included 18 patients who were homozygous wt/wt and twelve subjects who were heterozygous for the wild type and variations of either *4 or *5. Comparisons of ASEX total scores and subscale scores between genotype groups did not result in significant findings. When CYP2D6 metabolic activity was assessed using dextromethorphan measures, those classified as having a PM *phenotype* reported significantly higher rates of anorgasmia (males and females) and impaired lubrication (females only) than those with normal CYP2D6 metabolic activity. Differences between the EM and PM groups on the erection/ lubrication item reached statistical significance (*p* = 0.007), as did the lack of orgasm item (*p* = 0.009). The attitudes of subjects toward SD during paroxetine therapy were also assessed. SD was expressed as an undesirable complication by 12/30 (40%) of participants. This study was limited by the small sample size and the non-comprehensive genotyping strategy used to characterize CYP2D6 metabolizer status by genotype. Using a phenotyping method, the authors identified associations between metabolizer status and some measures of the ASEX scale. Notably, the metabolizer status observed in these participants was likely a function of paroxetine exposure, which is a potent CYP2D6 inhibitor [19]. Therefore, “metabolizer” phenotype status may have been a proxy for a combination of paroxetine exposure and genetics. Conclusive explanations of these findings warrant further investigation. Nonetheless, this small study is one indicator that drug metabolism and dose response may be an important consideration in the development of SSRI-associated sexual side effects. 

The second study of *CYP2D6* pharmacogenetics was also completed by Zourkova *et al.* [22] as a follow up to the previous investigation. This was a longitudinal study of 55 outpatients with a diagnosis of either depression (n = 23) or an anxiety disorder (n = 32). Seventeen males and 38 females were treated with paroxetine 10-40mg/d dosed in a flexible manner for a period of 2-16 months. Subjects were assessed using the Clinical Global Impression-Severity of Illness Scale (CGIS) and the Arizona Sexual Experiences Scale. CYP2D6 phenotyping was completed as previously outlined (Zourkova 2002). Genotyping for CYP2D6*3, *4, *5, and *6 variants was done to investigate genotypes associated with reduced CYP2D6 function. Women were more likely to develop SD than men in this population. Approximately 41% of men and 81% of women were categorized has having sexual dysfunction as defined by ASEX total scores exceeding 19. Women had greater ASEX scores on all of the subscale measures that were also assessed in males. Separating ASEX scores by genotype groups did not result in significant differences. When CYP2D6 metabolizer status was determined using dextromethorphan *phenotyping* procedures, a total of seven men and 12 women were assigned to the EM phenotype group, while 26 women and 10 men were categorized as PMs. ASEX total scores and subscale scores did not differ in males across EM and PM groups. However, female PMs had higher (worse) total, satisfaction, orgasm, and lubrication scores than EMs. Similar to the first study by this research group, the genotyping methods and sample sizes were likely inadequate for assessing the relationship between genetically derived PM status and paroxetine-associated sexual dysfunction. However, the PM *phenotype* differences may be a proxy for paroxetine exposure and support previous evidence that sexual dysfunction may be a dose-dependent phenomenon.

### 3.3. Pharmacogenetic Studies of SSRI-Associated Sexual Dysfunction: Genetic Variation in Genes Related to SSRI Pharmacodynamics

Initial studies investigating variations in genes related to the pharmacodynamics of SSRIs are yielding promising results while providing insight on additional pathways and variables for further study. In 2006, Bishop *et al.* published the first investigation of the relationship between functional polymorphisms in the genes hypothesized to influence SSRI signaling and SD [23]. Gene variants assessed included the *HTR2A* -1438 G/A (rs6311) single nucleotide polymorphism (SNP) in the *HTR2A* receptor gene and the C825T (rs5443) SNP of the G-protein beta-3 subunit gene (*GNB3*), which codes for a G-protein second messenger complex associated with 5HT2A receptor signaling. The -1438A variant exhibits greater gene expression in cell lines which express endogenous 5HT2A [24] and the genetic variation in *GNB3T* allele is thought to increase second messenger signaling [25,26]. 

This point prevalence study included 89 outpatients between the ages of 18 and 40 years with depression who had been treated with an SSRI for at least six weeks and had not experienced problems with sexual functioning before starting treatment. The investigators excluded individuals with concomitant disease states known to influence sexual well-being (e.g. hypertension, diabetes, *etc.*) as well as potential participants taking any other medication besides an SSRI known to positively or adversely influence sexual function. Subjects were recruited if they had been taking an SSRI for at least six weeks to minimize the confounding effects of depressive symptoms. Participants were assessed with clinical ratings of depression and anxiety, and the Changes in Sexual Functioning Questionnaire (CSFQ), which is a validated measure of global sexual function as well as several aspects of sexual well-being (e.g. satisfaction/pleasure, arousal, orgasm, desire, and frequency) [27]. The primary outcome variable of this study was a global measure of SD, defined as exceeding predefined sex-specific thresholds on CSFQ total scores. Secondary outcomes included CSFQ subscale measures. Mean Hamilton Depression Rating Scale (HAM-D) and Hamilton Anxiety Rating Scale (HAM-A) scores were <7, indicating that these patients had largely responded to treatment at the time of assessment. SSRI-related SD in this study population had an overall prevalence of 31% (35% in females and 18% in males). After controlling for age, sex, anxiety scale scores, and depression scale scores, subjects with a GG genotype of the *HTR2A* _1438 SNP were more likely to be categorized as having SD than those with a GA or AA genotype (OR = 3.6; p = 0.046). In addition, the *HTR2A* _1438 GG genotype predicted lower arousal scores (p = 0.022). The authors concluded that the *HTR2A* rs6311 SNP is an important predictor of SD in patients who were clinically asymptomatic and at otherwise low risk of sexual side effects.

A follow up study by Bishop *et al.* was conducted to investigate variants in the serotonin transporter gene (*SLC6A4*) and their relationships to SSRI-associated SD [7]. Two common functional variations were assessed, the *SLC6A4* promoter region (5HTTLPR) insertion/deletion variant and a variable number of tandem repeats (VNTR) in the second intron which are both associated with expression levels and function of serotonin transporters [28,29,30]. The *HTR2A* -1438A/G variant was also included in *post hoc* analyses to confirm previous results. This study included 115 subjects aged 18-40 years with identical inclusion/exclusion criteria and assessments as detailed in the aforementioned publication [23]. The primary outcome variable as before was exceeding the sex-specific thresholds for SD on the CSFQ total scale scores with arousal and orgasm subscale measures assessed as secondary outcomes separately in males and females. The primary finding of this study was that the 5HTTLPR insertion/deletion L allele was associated with SD (OR = 2.7; p = 0.02). The relationship between promoter genotypes and sexual well-being differed in men and women, and was dependent on whether or not women were taking an oral contraceptive (OC) medication. Females with the LL genotype of the 5HTTLPR who were taking OCs were nearly eight times more likely to be categorized as having SD, while no correlation was observed in women who were not taking OCs. The authors concluded that the serotonin transporter insertion/deletion is associated with sexual complications in persons being treated with SSRIs. This relationship may differ by sex, and may depend on OC status in women. 

Zou *et al.* assessed the relationship between a SNP in the brain-derived neurotrophic factor gene (*BDNF*) and sexual side effects as one outcome in a pharmacogenetic study of fluoxetine for the treatment of major depressive disorder in Chinese subjects [31]. BDNF is hypothesized to be involved with the underlying pathophysiology of depression [32]. The *BDNF* gene encodes a precursor peptide that is proteolytically cleaved to create the active BDNF protein. The human *BDNF* gene contains a functional 196G/A single nucleotide polymorphism (rs6265), which induces amino acid substitution from valine to methionine in exon 1 (Val66Met) and alters this cleavage site. Three hundred and five Chinese patients suffering from major depressive disorder (MDD) were included in this pharmacogenetic study which was a secondary analysis of a larger (n = 1038) investigation of the effects of fluoxetine for the treatment of MDD. The subjects genotyped for *BNDF* Val66Met were over 18 years of age, had experienced depressive symptoms for at least two weeks prior to the start of the trial, and had not been treated with medication for depression or undergone electroconvulsive therapy during the prior six months. Participants were administered 20 mg/day fluoxetine for six weeks. They were evaluated at baseline and after weeks 1, 2, 4 and 6 using the 17-item HAM-D and the Treatment-Emergent Symptoms Scale (TESS). The study reported no evidence for association between the *BDNF* Val66Met polymorphism and the efficacy of fluoxetine. However, the *BDNF* Met allele carriers had a lower incidence of decreased sexual desire (OR = 0.32, p = 0.01) as compared to the Val/Val genotype group. The authors controlled their analyses for age, sex, height, weight, age at onset, baseline HAM-D scores, smoking status, alcohol consumption and treatment history. 

The largest pharmacogenetic study of SSRI-associated sexual side effects was recently completed in the Sequenced Treatment Alternatives to Relieve Depression (STAR*D) clinical cohort. STAR*D is the largest effectiveness trial of antidepressant treatment to date [4] and is providing important insight into treatment response profiles in a large “real world” study sample of subjects with MDD. The initial phase, which served as the study sample investigated here, was comprised of subjects with MDD who were treated with the SSRI citalopram in a flexible dosed manner up to 60 mg/d for up to 14 weeks. The entire investigation included 4,041 subjects, 1,910 of whom provided blood samples and were included in genetic and pharmacogenetic analyses [14]. The primary goal of this particular analysis was to investigate the relationship between serotonergic, glutamatergic, dopaminergic, adrenergic, and neurotrophic candidate genes and sexual side effects as measured by the Patient-Rated Inventory of Side Effects (PRISE) scale. The 768 genetic variants selected were also studied in other genetic/pharmacogenetic studies of SSRI treatment response and SSRI-associated suicidal ideation [33,34,35,36]. Of the 1,910 subjects assessed, 1473 (78.5%) were classified as Caucasian. To minimize the confounding effects of ancestry, only the results from Caucasian subjects were included in the analysis. The subjects included in these studies were 39% male, 42.7 ± 13.5 years of age, and had a mean duration of illness of approximately 16 years. Symptom response to treatment was measured with the Quick Inventory of Depressive Symptomatology (QIDS-C_16_) scale. Primary outcomes assessed from the PRISE scale included libido (all subjects), orgasm (all subjects) and erectile dysfunction (males only). 

A significant proportion of participants reported decreased libido (54%) and orgasm difficulties (36%). Approximately 37% of men reported erectile dysfunction. Analyses conducted by these investigators assessed associations between genetic markers (individual SNPs, SNPs grouped by gene, and genetically determined population substructure) as well as clinical variables and the sexual dysfunction outcomes listed above. Clinical variables associated with the primary outcomes were included as covariates in secondary logistic regression analyses to determine whether they influenced any genetic relationships that were discovered. For erectile dysfunction this included increased age, duration of illness, severity of depression, cumulative medical comorbidities, and diagnosis of an anxiety disorder. Decreased libido was associated with earlier onset of illness, severity of depression, being married, and being male. Orgasm dysfunction was associated with younger age, early onset of depression, cumulative medical comorbidities, being married, and being male. Subjects included in these analyses were those who provided DNA, were prescribed citalopram, and returned for at least one follow up visit. Due to the broad inclusion and exclusion criteria of the STAR*D study, subjects may have been taking other non-psychotropic medications or had concomitant medical illness that may have influenced sexual function. Associations were examined regardless of whether depressive symptoms improved after treatment. Additionally, subjects were not assessed with the PRISE before treatment with citalopram, therefore baseline assessments of sexual functioning were not available for study. Of the 768 SNPs studied across 68 different candidate genes, the authors identified significant associations between variants in the *GRIK2*, *GRIA1*, and *GRIN3A* genes with sexual side effect assessments included on the PRISE. Decreased libido was associated with four different SNPs in the *GRIK2* gene (rs51326, rs9404130, rs2518302, rs2518244), and three SNPs in *GRIA3* (rs2285127, rs2269551, rs550640). Three SNPs in *GRIA3* (rs1994862, rs10515697, rs1864205) were associated with orgasm difficulties. Finally, three SNPs in *GRIN3A* (rs1323427, rs1323423, rs2050641) were associated with ED in males. With respect to other results summarized in this review, the authors investigated the relationships between previously associated SNPs (e.g., *HTR2A* rs6311) and sexual well-being outcomes in this cohort and did not observe evidence for a relationship in this study sample. Additionally, while not explicitly described in this analysis, previous studies of this cohort did not find significant associations of *BDNF* variants with response to citalopram [35]. This highlights the potential influence that differences in the assessment methods used, comorbidities of the subject populations, and depressive symptoms may have on the results of pharmacogenetic association studies. Nevertheless, these results indicate that glutamate system genes may warrant further investigation in pharmacogenetic studies of SSRI-associated SD.

### 3.4. Pharmacogenetic Studies of SSRI Treatments for Sexual Dysfunction

The topic of SSRIs and SD generally centers on this spectrum of side effects being *caused* by antidepressants. In addition to lack of desire and sexual function and performance difficulties, SD may also include premature ejaculation for males, which is considered a neurobiological disorder with genetic predisposition [37]. To this end, one pharmacodynamic effect of SSRIs in males (e.g., delayed orgasm) that is a *side effect* in patients being treated for depression or other disorders is considered a *treatment* for patients with a condition known as lifelong premature ejaculation (LPE). LPE is characterized by consistent intravaginal ejaculation latency times (IELTs) of less than 1 minute and is associated with disturbances of the serotonin system [37]. Premature ejaculation (PE) is the most common form of male SD, and the prevalence of PE varies depending on the study sample assessed. Currently, the most widely accepted pharmacotherapeutic approach to treating PE is off-label administration of SSRIs including paroxetine, fluoxetine, sertraline, citalopram, and escitalopram. Although they are generally regarded as effective in treating PE, therapeutic response to SSRIs for this condition differs from patient to patient. One pharmacogenetic study has been conducted to assess the relationship between functional variation in the serotonin transporter gene (*SLC6A4*) and changes in IELT after SSRI treatment [38]. These genetic relationships with increased IELTs for the treatment of LPE may be informative for identifying underlying genetic contributors for anorgasmia or delayed orgasm in males being treated with SSRIs for mood or anxiety disorders.

To date, Safarinejad [38] has published the only pharmacogenetic study of an SSRI for the treatment of LPE. Two-hundred and forty-six men, all married and aged 20 to 42 years, participated in a fixed- dose treatment study of sertraline. Subjects had a diagnosis of LPE as previously defined and received 50 mg of sertraline daily for two weeks, followed by 100 mg daily for 10 more weeks. Participants utilized a stopwatch method for quantifying IELT, with change in IELT from baseline to 12 weeks used as the primary outcome variable. Subjects were genotyped for the *SLC6A*4 5HTTLPR polymorphism along with a variable number of tandem repeats (VNTR) variant in the second intron (STin2), which has previously been reported to function as a transcriptional regulator of serotonin transporterexpression [39]. Allelic and genotype associations with change in IELT over time were assessed. Overall, of the 227 participants 165 (72.7%) responded to treatment, as indicated by an IELT greater than 1 minute. IELT improvements over time differed significantly by *SLC6A4* genotypes. Subjects who were carriers of an “L” allele of the 5HTTLPR had a greater response to sertraline. The authors report that subjects with the L/L genotype were more likely to respond to sertraline therapy than those with other genotypes. Furthermore the STin210/10 genotype was more commonly observed in non-responders, a finding that further supports the relationship of variation in the serotonin transporter gene with SSRI pharmacodynamics.

## 4. Discussion

SSRIs are often chosen as first-line pharmacological treatments for depression. These drugs are generally better tolerated and are less likely to induce toxicity than older agents such as tricyclic antidepressants (TCAs) and monoamine oxidase inhibitors (MAOIs) [2,3]. Unfortunately, sexual dysfunction is commonly observed during SSRI treatment. These side effects are often long-lasting and resistant to treatment [9,10]. Common symptoms of SSRI-associated sexual dysfunction include decreased libido, problems with arousal and erection, and delayed or absent orgasm. The severity and presentation of these side effects varies greatly from patient to patient. In addition to depressive symptoms, drug dose, and other variables, genetic factors are now recognized as potential predictors of changes in sexual well-being while taking this class of medications. 

Initial pharmacogenetic studies of SSRI-associated sexual dysfunction are comprised largely of candidate gene association investigations characterizing the relationships of serotonin, glutamate and drug metabolism genes with SSRI-associated sexual dysfunction. The sexual domains studied include libido, sexual arousal, erectile dysfunction, and anorgasmia. Additional insight into the effects of SSRIs on the physiology of the sexual experience is provided by studies of SSRIs for the treatment of premature ejaculation and associations of variations in the serotonin transporter with response/non-response to these treatments. 

The elusive nature of sexual dysfunction makes it challenging to assess the role of antidepressant effects on the sexual experience; isolating antidepressants as the single contributing factor is not always straightforward. Thus, important aspects of evaluating the studies completed to date include study design and the methods for assessing sexual functioning. Three of the six studies of SSRI-associated sexual dysfunction were prospective, while three were point-prevalence/cross-sectional investigations. However, two of the point-prevalence/cross-sectional studies had narrowly defined inclusion/exclusion criteria and utilized the CSFQ assessment, which is a measure validated for use in the populations examined [7,23]. These studies also excluded potential subjects with concomitant disease states or drug utilization that may have confounded the evaluations of sexual well-being which were the primary goals of these investigations. To date, prospective studies have utilized shorter assessments or extracted sexual functioning questions from larger, more general side effect questionnaires, which are part of commonly used rating scales used in large clinical trials. The prospective studies, which assessed sexual well-being in secondary analyses, were less restrictive in the exclusion of participants with other disease states or confounding medications. While these populations may be more representative of patients encountered in clinical practice, confounding disease states and medications along with assessments not specifically validated for sexual functioning assessments may be reasons for discrepant results across studies. 

Studies to date are inconclusive regarding the relationship between drug metabolism and sexual dysfunction [21,22]. The studies which did attempt to investigate this relationship were inadequately powered to detect the effects of CYP2D6 poor metabolizer status and paroxetine-associated sexual side effects. Larger studies indicate that a dose-response relationship between some aspects of sexual dysfunction may exist [8,19]. Thus, it follows that patients who are poor metabolizers for CYP2D6 or CYP2C19, which are two essential enzymes responsible for the metabolism of SSRIs, may be at greater risk for sexual side effects at a given dose. Pharmacogenetic studies assessing sexual side effects have not been fixed dose investigations. In the context of this type of study design, doses may be titrated to tolerability which limits our ability to determine whether drug metabolism variations are important risk factors for sexual side effects.

Candidate genes hypothesized to be related to the pharmacologic mechanisms of SSRI action or the pathophysiology of depression have yielded initial insight into potential genetic correlates of sexual side effects from SSRIs. Results from analyses of the -1438 (rs6311) SNP in the *HTR2A* gene suggest that genotypes associated with lower transcription of the 5HT2A receptors in cell culture experiments [24] are more sensitive to decreases in arousal associated with SSRI exposure [23]. The mechanisms of these effects are unknown. It is plausible that individuals with lower baseline expression levels of post-synaptic 5HT2A receptors may be more susceptible to receptor saturation and enhanced signaling through this pathway which is hypothesized to be important for modulating arousal and sexual function [13]. A larger analysis conducted by Perlis *et al.* [14] did not find an association in the STAR*D cohort, although whether this is a result of differences in the study population assessed, the nature of the questionnaire used for these outcomes in STAR*D, or type-1 error in previous studies is uncertain. Thus, further research needs to be done to better characterize this relationship. However, these studies highlight the importance that study design and phenotype measure differences may influence the results observed.

Two studies highlight the importance of the commonly studied serotonin transporter promoter region insertion/deletion and SSRI effects on sexual well-being. Bishop *et al.* identified an association between the “L” genotype and an increased likelihood of experiencing sexual dysfunction as measured on CSFQ totals scores [7]. Further analyses identified that this relationship appears to be more robust in females who are also taking estrogen-containing oral contraceptives. The results of Safarinejad [38] are consistent with these findings in that subjects with an LL genotype were most likely to respond (increase in IELT) to SSRI therapy for premature ejaculation. The study by Bishop *et al.* did not observe evidence for association in males when the results were stratified by sex, although their study was not adequately powered to detect these effects in males. It is plausible that the underlying mechanism of these effects may be related to the increased expression of serotonin transporters observed in the context of the LL genotype. Although this mechanism remains to be elucidated, serotonin transporter antagonism by SSRIs increases serotonin transmission through postsynaptic serotonin receptors including 5HT2A. Patients with greater transporter expression (e.g. those with an LL genotype) prior to treatment may exhibit an increase in signaling at these postsynaptic receptors, which is consistent with data suggesting that these patients have a greater clinical improvement to SSRI therapy [40]. However, other pharmacogenetic investigations have associated the “S” allele or “SS” genotypes with lower tolerability to SSRIs [41,42], although the side effect dimensions studied did not explicitly include assessments of sexual function.

Preliminary data from an analysis of the *BDNF* gene are also indicative of the important role of genetic variation in the serotonin system in sexual functioning [31]. Zou *et al.* reported that the *BDNF* Met genotype was associated with a lower risk of sexual side effects and insomnia in a large study of Chinese subjects prospectively treated with fluoxetine. The potential mechanism underlying this relationship is unclear. However the interaction between genetic variation in the serotonin transporter and this variation in *BDNF* has been described [43,44,45] to influence depressive symptoms and personality traits. Although this interaction was not assessed by Zou *et al.* it is plausible that this *BDNF* polymorphism may also be indirectly or directly related to sexual well-being and that modulation of this system by SSRIs like fluoxetine differentially influences the likelihood of experiencing sexual side effects.

The recent study by Perlis *et al.* provides interesting information about the potential role of the glutamate system with SSRI-associated changes in sexual functioning [14]. These authors describe associations between *GRIA3* and *GRIK2* and decreased libido, *GRIN3A* and erectile dysfunction, and *GRIA1* and delayed or absent orgasm. The functional consequences of the variants associated in the largest pharmacogenetic analysis of sexual dysfunction to date are unknown. Further, the role of the glutamate system in the sexual experience and SSRI mechanism of action is unknown. A previous analysis of the STAR*D sample describes associations between glutamate system genes and citalopram-associated suicidal ideation and depressive symptoms [36]. However, the sexual dysfunction analysis accounted for clinical presentation and depressive symptoms in their analysis indicating that further characterization of the relationships between these genes and sexual well-being during antidepressant treatment is warranted. 

## 5. Conclusions

Sexual side effects from SSRI antidepressants are common, persistent, and vary in intensity and presentation across patients. Initial studies characterizing the contribution of genetic variability and SSRI-associated changes in sexual function provide important insight into the potential for pharmacogenetic information to influence drug selection for depression and other disorders treated with SSRIs. While requiring further mechanistic clarification and replication, variants in serotonin genes (*HTR2A* and *SLA6A4*), a gene interacting with the serotonin system (*BDNF*) as well as glutamate system genes (*GRIK2, GIRA3, and GRIA1*) appear to be associated with SSRI-associated sexual dysfunction. In some cases, the nature of these relationships appears to differ in males and females as well as the domain of sexual function studied. The importance of study design and methods of assessing sexual function are important and heterogeneity in these aspects across studies makes direct comparison of results across investigations difficult. Nonetheless, these initial pharmacogenetic studies of SSRI-associated sexual dysfunction will hopefully set the stage in this area for future research that will determine the potential for this information to be translated to clinical care.
